# Health disparities in the detection and prevalence of pediatric obstructive sleep apnea

**DOI:** 10.3389/frsle.2023.1225808

**Published:** 2023-08-10

**Authors:** Maureen E. McQuillan, Ian C. Jones, Haneen F. Abu Mayyaleh, Shajna Khan, Sarah M. Honaker

**Affiliations:** Indiana University School of Medicine, Indianapolis, IN, United States

**Keywords:** pediatric obstructive sleep apnea, health disparities, PSG (polysomnography), insurance, race, ethnicity, socioeconomic status

## Abstract

**Introduction:**

Pediatric Obstructive Sleep Apnea (OSA) is associated with negative health outcomes, behavior problems, and poor academic performance when left untreated. Prior research has shown that children from racial and ethnic minority backgrounds and those living in lower socioeconomic status (SES) homes or neighborhoods have higher prevalence of OSA but lower likelihood of receiving evidence-based care for OSA. Disparities in pediatric OSA detection (e.g., timely assessment and diagnosis) likely contribute to this observed inequity in receiving treatment. A polysomnogram (PSG) is the gold standard for diagnosing OSA but completing PSG can be challenging. Study aims were to examine racial, ethnic, and SES differences in (1) OSA prevalence and severity and (2) OSA detection, specifically PSG completion rates, timing since referral, and age of diagnosis.

**Methods:**

Children (aged 1–18, *N* = 1,860, 56% male) were referred for PSG during a 6-month period. Participants' racial/ethnic background were as follows: 64.8% White non-Hispanic, 23.5% Black non-Hispanic, 9.4% White Hispanic, and 2.4% other. Children predominantly had Medicaid insurance (64.5%). SES was measured by insurance type and neighborhood SES using the Distressed Communities Index (DCI) for each participant's zip code (Economic Innovations Group; https://eig.org/dc). Covariates included child age and sex, BMI, premature birth status, and smoke exposure in the home.

**Results:**

We replicated previous research by showing that children from minority racial/ethnic backgrounds and lower SES backgrounds had higher prevalence rates of OSA and worse disease severity. Across racial, ethnic, and socioeconomic backgrounds, only 31.6% of the children referred successfully completed PSG. Insurance coverage (Medicaid or private vs. self-pay), was an important factor in predicting earlier timing and better completion rates of PSG, which is essential for successful diagnosis and treatment of pediatric OSA.

## Introduction

Obstructive sleep apnea (OSA) is a sleep disorder involving obstruction of the upper airway, which disrupts ventilation and sleep patterns (ICSD, 2014). The primary cause of pediatric OSA is anatomical obstruction due to large tonsils and adenoids (Katz and D'Ambrosio, 2008). Untreated pediatric OSA has been associated with higher levels of behavior problems, including parent-reported inattentiveness, hyperactivity, aggression, and rule breaking (Csábi et al., 2022), negative health outcomes (Thomas et al., 2022), and poor academic performance (Harding et al., 2020). Given these negative outcomes, timely diagnosis and treatment is critical. Polysomnography (PSG) is the gold-standard for diagnosing OSA, and a tonsillectomy and adenoidectomy (T&A) is the first line treatment for pediatric OSA (Marcus et al., 2012), as it is curative in up to 80% of cases (Oguh et al., 2022).

### Disparities in OSA prevalence

Racial, ethnic, and socioeconomic disparities in pediatric OSA prevalence rates have been well-established (Williamson et al., 2023). For example, children who are classified as Black or Hispanic are more likely to have OSA, and to have more parent-reported, related symptoms of sleep disordered breathing and daytime sleepiness (Goodwin et al., 2003; Kilaikode et al., 2018). Black children have also been shown to have more severe OSA, based on having higher Apnea Hypopnea Index (AHI) scores (Weinstock et al., 2014). Similarly, children from lower income areas are more likely to have OSA (Spilsbury et al., 2006; Brouillette et al., 2011), and children whose mothers have lower levels of educational attainment have been shown to have higher prevalence and greater severity of OSA (Park et al., 2022). Moreover, some children face compounded risk factors—for example, Black children are more likely to have Sickle Cell Disease (SCD), and individuals with SCD are more likely to also have OSA (Tsou et al., 2021).

### Disparities in OSA detection and treatment

Inequities in OSA detection and treatment have also emerged. For instance, Black children have been shown to have longer time to OSA diagnosis (Kilaikode et al., 2018**)** and to receive T&A for OSA at half the frequency of White peers (Morton et al., 2001). Regarding socioeconomic disparities, families below the federal poverty level who are eligible for public insurance (i.e., Medicaid) are less likely to receive a referral for a T&A, with providers citing reasons of poor reimbursement and excessive paperwork (Wang et al., 2004). These publicly-insured children also tend to experience longer intervals from referral to PSG to treatment (Boss et al., 2011), compared to those who are privately insured. Moreover, in a sample of over 80,000 children on Medicaid, only 15.4% of children with sleep disordered breathing received T&A, across racial and ethnic backgrounds (Pecha et al., 2021).

### The present study

Pediatric OSA can be reliably detected and treated with PSG and T&A, but if it is left undiagnosed and untreated, children face a number of negative socioemotional, physical, and academic outcomes. Children from under-represented racial, ethnic, and socioeconomic backgrounds may be the most at-risk for these negative sequela as they are the most likely to have OSA and the least likely to receive OSA treatment. Successful PSG completion rates after referral, and potential health disparities involved in following through on this referral, have been understudied. Once a child is referred for PSG, families may face difficulties navigating the referral and scheduling process, as well as other barriers, such as transportation, childcare for other siblings, and health insurance to cover the cost of the study. If PSG completion and OSA detection are delayed or not completed because of such barriers, children may be older at the time of diagnosis, may experience more severe OSA, with higher AHI values, and more unmitigated outcomes.

### Aims and hypotheses

The present study aimed to (1) examine racial, ethnic, and socioeconomic disparities in OSA rates and disease severity, and (2) evaluate whether there were inequities in timely OSA detection by examining PSG completion rates, PSG timing after referral, and child age at the time of the PSG. We hypothesized that children from lower socioeconomic backgrounds and those classified as underrepresented minorities (URMs) would be more likely to be diagnosed with OSA, have more severe OSA (with higher AHI values, and more sleep disordered breathing symptoms, and daytime sleepiness), and that these children would be less likely to complete PSG, have a longer duration of time between referral and PSG, and/or complete the study or receive an OSA diagnosis at an older age compared to peers.

## Methods

### Procedure

The study involves retrospective analysis of de-identified clinical information provided by pediatric patients. Electronic health records of all children referred from January 2019 through June 2019 for diagnostic PSG for the indication of sleep-disordered breathing were retrospectively reviewed. Referrals came from pediatric primary care providers and specialists, and all referrals were for the sleep lab at the children's hospital at our academic medical center. PSG completion rates and service dates, along with diagnostic and demographic information, were collected. Parents also completed sleep questionnaires to provide parent-report of children's sleep disordered breathing symptoms and daytime sleepiness (when age appropriate). These questionnaires were either completed before the sleep study (for families that completed PSG) or during a clinic visit to the sleep center, as part of routine clinical practice. This study involving human participants was reviewed and approved as exempt by the Indiana University School of Medicine Institutional Review Board. Written informed consent from participants was waived and not collected; thus, we have removed any identifiable information by only providing age ranges of participants.

### Participants

Electronic health records were examined for 1,860 participants (aged 1–18, *M* = 6.12, *SD* = 4.40 years, 56% male) who were referred for a sleep study. Participants were predominantly White non-Hispanic (64.8%), and the children predominantly received public insurance (i.e., Medicaid; 64.5%). Complete demographic information is listed in Table 1.

**Table 1:** Sample demographics.

	***N (%)***
# Children referred	1,860 (100)
Completed PSG	587 (31.6)
Diagnosed with OSA	277 (14.9)
**Child sex**	
Male	1,049 (56.4)
Female	811 (43.6)
**Racial/ethnic backgrounds**	
White, non-Hispanic	1,205 (64.8)
Black, non-Hispanic	437 (23.5)
White, Hispanic	174 (9.4)
Other	44 (2.4)
**Maternal education**	
Less than high school degree	130 (6.8)
High school diploma/GED	558 (30.1)
Some college	707 (37.7)
Bachelor's degree and beyond	465 (25.4)
**Insurance type**	
Public (Medicaid)	1,200 (64.5)
Private	589 (31.7)
Self-pay	71 (3.8)
**Distressed communities index**	
Prosperous	351 (18.9)
Comfortable	299 (16.1)
Mid-tier	315 (16.9)
At-risk	261 (14.0)
Distressed	634 (34.1)

Percentages are based on the full sample of children who were referred for diagnostic testing (N = 1,860).

### Measures

#### OSA prevalence and severity

Among the 587 (31.6%) children who completed PSG, we recorded the AHI and whether an OSA diagnosis was made. The AHI is an index of the number of apneas (instances of total cessation of breathing) and hypopneas (instances of partial cessation of breathing) per hour of sleep. A higher AHI indicates greater severity of disease. Consistent with the clinical guidelines used at our sleep center, an AHI >1.5 indicates the presence of OSA in children. In this study, we classified whether or not an OSA diagnosis was made based on the physician's summary in the child's PSG report, and the physicians used this scoring criteria to make a diagnosis. Among the referred children who completed PSG, the average AHI was 5.61 (*SD* = 11.15). In terms of severity in our sample of children who completed PSG, 46.34% had AHI values that were within normal limits (< 1.5), 29.13% had mild OSA (1.5 < AHI < 5), and 24.53% had moderate to severe OSA (AHI > 5).

#### Symptom severity

Symptoms related to OSA, specifically sleep disordered breathing and excessive daytime sleepiness, were also examined. The Children's Sleep Habits Questionnaire (CSHQ) has a subscale for sleep disordered breathing assessing parent-reported frequency of loud snoring, pauses in breathing, and snorting/gasping from “rarely” (0-1x/week) to “usually” (5-7x/week; Owens et al., 2000). The sleep disordered breathing subscale score was used to assess sleep disordered breathing symptoms (*M* = 5.69, SD = 1.96).

The Epworth Sleepiness Scale (ESS) is an eight-item questionnaire assessing the likelihood of falling asleep in eight common situations, such as while riding in a car (Johns, 1991). The total score of the ESS ranges from 0 to 24. In clinical practice, a score of ten is a frequently-used cut-off to denote excessive daytime sleepiness. The ESS was initially designed for adults and then was modified for use with school-aged children and adolescents (Moore et al., 2009). The sum was used in all analyses (*M* = 7.83, SD = 5.29). The ESS was only administered in school-aged children and adolescents (i.e., participants ages 7 and above), as it has not been validated in younger children and would not be developmentally-appropriate for young children, many of whom still take regular daytime naps.

#### Race/ethnicity

To increase power and reduce the number of statistical tests, we collapsed the racial and ethnic groups presented in Table 1 down to White, non-Hispanic and underrepresented racial and ethnic minorities (URMs), which included those who identified as Black Non-Hispanic, White Hispanic, and Other. As a sub-analysis, we also present the data for participants identified as White, non-Hispanic compared to Black, Non-Hispanic and White, Hispanic for each aim.

#### Socioeconomic variables

Insurance type, either Medicaid, Private, or Self-pay/Uninsured, was collected from the electronic health record. The Distressed Communities Index (DCI) was used as another SES measure. The DCI is composed of seven variables, including high school graduation rate, poverty rate, unemployment rate, housing vacancy rate, median household income, change in the number of jobs available over the past 5 years, and change in the number of businesses in the area for the past 5 years, for each zip code (Economic Innovations Group; https://eig.org/dci). The DCI ranks zip codes based on their relative economic performance on these seven key metrics to determine economic distress at the zip code level, ranging from 0 to 100, where higher scores reflect more distress. The DCI can be further categorized into five groups: prosperous, comfortable, mid-tier, at risk, and distressed. Both the continuous average and the categorical classifications were used.

### Analysis plan

For the first aim, we examined racial, ethnic, and socioeconomic disparities in OSA diagnosis rates using chi-square tests. We also examined differences in disease severity by comparing group means for AHI values and related OSA symptoms (i.e., sleep disordered breathing symptoms and daytime sleepiness) using *t*-tests. For the second aim, we examined racial, ethnic, and socioeconomic differences in PSG completion rates and PSG timing. For PSG timing, we used the number of months between the date of referral and date of service. We also analyzed timing differences by examining child age at the time of PSG completion and child age at the time of diagnosis (for those who were diagnosed with OSA). Age at PSG and age at diagnosis were equivalent, as they were both based on the age of the child at the time of the sleep study.

For all analyses, we controlled for child sex, BMI, premature birth status, and smoke exposure in the home. BMI was recorded at the time of PSG. We recorded both BMI raw scores and percentiles, accounting for child age. Premature birth status and smoke exposure in the home were both provided via parent report in the questionnaire that was administered either before the sleep study or during routine clinical care (along with the CSHQ and the ESS). We also controlled for SES when analyzing the role of race and ethnicity, and vice versa. For a sensitivity analysis to evaluate the impact of age, we examined whether disease prevalence, severity, and detection differed by child age by categorizing the children into three age groups—those less than 8 years old, 8–12.99 years old, and 13 years and older.

## Results

### Aim 1. Disparities in OSA prevalence and severity

Of the 1,860 children referred for a sleep study, 277 (14.9%) were diagnosed with OSA, which is 47.2% of those who completed PSG. On average, the AHI was 5.61 (*SD* = 11.15) for those who completed PSG. Comparisons across racial and ethnic groups, child age, and insurance class are listed in Table 2.

**Table 2:** Disparities in OSA prevalence and severity.

**Group**	**Rate of OSA dx *%***	**AHI *M (SD)***	**SDB *M (SD)***	**ESS *M (SD)***
Full sample (*N =* 1,860)	14.9	5.61 (11.15)	5.69 (1.96)	7.83 (5.29)
PSG completion sample (*N =* 587)	47.2	5.61 (11.15)	5.83 (2.02)	7.93 (5.22)
White, non-Hispanic	45.0	4.56 (9.09)	5.51 (1.92)	7.38 (4.99)
URM	**50.9** ^**∧**^	**7.44 (13.88)** ^*^	**6.08 (2.01)** ^*^	**8.83 (5.78)** ^*^
< 8 years old	47.1	4.65 (8.54)	5.87 (1.99)	7.12 (4.37)
8–13 years old	51.5	5.68 (10.29)^∧^	**6.13 (2.00)** ^*^	9.07 (6.35)
13+ years old	41.7	**10.97 (20.44)** ^*^	5.22 (2.18)	**9.71 (6.09)** ^*^
Medicaid	49.0	**6.39 (12.61)** ^*^	5.75 (1.97)	8.06 (5.32)
Self-pay	64.3	4.99 (5.34)^*^	5.90 (1.79)	8.50 (5.47)
Private	41.8	**3.90 (7.14)** ^*^	5.54 (1.97)	7.39 (5.20)

OSA, Obstructive Sleep Apnea; dx, diagnosis; AHI, Apnea Hypopnea Index, a measure of disease severity; SDB, the sum of parent-reported sleep disordered breathing symptoms; ESS, Epworth Sleepiness Scale, parent-report measure of daytime sleepiness; PSG, Polysomnogram; URM, Children who were classified as underrepresented racial and ethnic minorities.

^∧^p < 0.10.

^*^p < 0.05.

For the rate of OSA diagnosis, the percentages do not equate to 100% because they should not be summed going down the rows. Each percentage is the percent of “X category” who was diagnosed with OSA. For example, in row 3 column 1, 45% means that 45% of the White, non-Hispanic participants who completed PSG were diagnosed with OSA, compared to 50.9% of the under-represented minorities who completed PSG were diagnosed with OSA.

The AHI values for the first two rows are equivalent as AHI could only be calculated on those who completed PSG. Significant findings are bolded.

#### Racial and ethnic disparities

Among all children who completed PSG, those who were categorized as from an under-represented minority background had higher rates of OSA diagnosis, at a level trending toward significance (50.9% vs. 45.0%, *X*^2^ = 1.89, *p* =.09). They also tended to have more severe OSA with higher AHI values (7.44 vs. 4.56, *t* = −3.03, *p* =.00), and to have higher scores for symptoms of sleep disordered breathing (6.08 vs. 5.51, *t* = −4.04, *p* =.00) and excessive daytime sleepiness (8.83 vs. 7.38, *t* = −3.34, *p* =.00), compared to peers who were classified as White non-Hispanic. The results were similar when children who were identified as White, non-Hispanic were compared to those identified as Black, non-Hispanic or White, Hispanic (see Table 3).

**Table 3:** Racial and ethnic disparities in OSA prevalence and severity.

**Group**	**Rate of OSA dx *%***	**AHI *M (SD)***
White, non-Hispanic	45.0	4.56 (9.09)
Black, non-Hispanic	**48.6** ^*^	**6.11 (10.98)** ^*^
White, Hispanic	**47.4** ^*^	**9.48 (19.45)** ^*^

OSA, Obstructive Sleep Apnea; dx, diagnosis; AHI, Apnea Hypopnea Index, a measure of disease severity; SDB, the sum of parent-reported sleep disordered breathing symptoms; ESS, Epworth Sleepiness Scale, parent-report measure of daytime sleepiness; PSG, Polysomnogram.

^∧^p < 0.10.

^*^p < .05.

For the rate of OSA diagnosis, the percentages do not equate to 100% because they should not be summed going down the rows. Each percentage is the percent of “X category” who was diagnosed with OSA. Significant findings are bolded.

#### Socioeconomic disparities

Although prevalence rates of OSA did not significantly differ fo**r** children who received Medicaid insurance vs. private insurance (49 vs. 41.8%), children who received Medicaid insurance had significantly more severe OSA, with higher AHI values (6.39 vs. 3.90, *t* = 2.46, *p* =.01), compared to children who had private insurance. DCI was not linked with either OSA prevalence (*r* = 0.02, *p* = 0.64) or severity *r* = 0.02, *p* = 0.62).

### Aim 2. Disparities in OSA detection

Five hundred eighty-seven of the referred children completed PSG, which is 31.6% of the total referred sample (*N* = 1,860). On average, it took children about 5.5 months to complete the diagnostic study, and children had an average age of 6.62 years (*SD* = 4.42 years) at the time of PSG. Comparisons across racial and ethnic groups, child age, and insurance class are listed in Table 4. There were no significant differences across racial and ethnic groups in terms of PSG completion, timing, or child age at the time of study or diagnosis, although children classified as from an under-represented minority background did tend to be slightly older, both at time of PSG and diagnosis, compared to White, non-Hispanic children. When we completed our sub-analysis, comparing children who were identified as White, non-Hispanic with those identified as Black, non-Hispanic or White, Hispanic, rather than collapsing them into the category of under-represented minorities, we again found no significant differences (see Table 5). There was only one exception – individuals identified as White, Hispanic appeared to complete PSG just under 1 month faster than individuals identified as White, non-Hispanic.

**Table 4:** Disparities in OSA detection.

**Group**	**Rate of PSG Completion *%***	**Time to PSG (m) *M (SD)***	**Age at PSG (y) *M (SD)***	**Age at Diagnosis (y) *M (SD)***
Full sample (*N =* 1,860)	31.6	5.52 (4.97)	6.62 (4.42)	6.47 (4.37)
White, non-Hispanic	31.0	5.63 (5.09)	6.45 (4.38)	6.35 (4.42)
URM	32.7	5.34 (4.74)	6.91 (4.47)	6.64 (4.30)
< 8 years old	NA	5.28 (4.87)	4.19 (2.28)	4.06 (2.34)
8-13 years old	NA	5.92 (4.88)	10.45 (1.05)	10.55 (1.14)
13+ years old	NA	6.33 (5.55)	15.01 (1.53)	14.80 (1.40)
Medicaid	33.0	5.57 (4.96)	6.59 (4.46)	6.45 (4.47)
Self-pay	**19.7** ^*^	6.79 (5.67)	**8.36 (5.88)** ^*^	6.60 (5.54)
Private	30.1	5.31 (4.94)	6.53 (4.18)	6.48 (3.98)

PSG, polysomnogram, the sleep study used to diagnose pediatric obstructive sleep apnea; URM, Children who were classified as underrepresented racial and ethnic minorities. Age groupings in the center-most column were based on when children completed PSG; Because all of these children completed PSG, rates of PSG are not applicable (NA). Significance testing was also not conducted between these age groupings since they were inherently separated by age. The right-most column showing age of diagnosis only pertains to the sub-sample of children who completed PSG and who were diagnosed with OSA (n = 277). Time to PSG is listed in months (m) and age at PSG and diagnoses are both listed in years (y).

^*^p < .05.

For the rate of PSG completion, the percentages should not equal 100 because each percentage is the percent of “X category” who completed PSG. For example, in row 2 column 1, 31% means that 31% of all the White, non-Hispanic participants referred for PSG actually completed PSG, compared to 32.7% of the under-represented minorities referred for PSG actually completed PSG. Significant findings are bolded.

**Table 5:** Racial and ethnic disparities in OSA detection.

**Group**	**Rate of PSG completion *%***	**Time to PSG (m) *M (SD)***	**Age at PSG (y) *M (SD)***
White, non-Hispanic	31.0	5.63 (5.09)	6.45 (4.38)
Black, non-Hispanic	32.0	5.56 (5.06)	6.69 (4.67)
White, Hispanic	32.8	**4.82 (4.14)** ^*^	7.33 (4.19)

PSG, polysomnogram, the sleep study used to diagnose pediatric obstructive sleep apnea; Time to PSG is listed in months (m) and age at PSG and diagnoses are both listed in years (y).

^*^p < .05.

For the rate of PSG completion, the percentages should not equal 100 because each percentage is the percent of “X category” who completed PSG. Significant findings are bolded.

Considering SES variables, children who were uninsured were the least likely to complete PSG (19.7% completion rate for families who were uninsured, compared to 33% for families who had Medicaid insurance and 30.1% who had private insurance, *X*^2^ = 6.38, *p* =.04). Relatedly, when the self-pay group completed PSG, these children tended to be older than their peers (8.36 years compared to 6.59 for children who had Medicaid insurance and 6.53 for children who had private insurance, *t* = 1.52, *p* =.04). Although there were only a few families in the sample who were both uninsured and completed PSG (*n* = 14), these children were also the most likely to be diagnosed with OSA (64.3%). The DCI score was not significantly linked with child age at time of PSG completion, *r* = 0.07, *p* = 0.08.

### Sensitivity analysis for child age

Table 2 shows that the oldest group of children, the adolescents, appeared to have more severe symptoms of OSA, with the highest AHI scores and ESS scores, compared to the younger children in the sample. Children who were aged 8–12 had the significantly highest scores for sleep disordered breathing symptoms.

When the three age groups were also examined across race/ethnicity (again White, non-Hispanic vs. URM), there were no significant differences for child age at PSG or child age at diagnosis. For example, among the adolescents, the average age at PSG for teens who were identified as White, non -Hispanic was 14.91 years and the average age at PSG for teens who were identified as an under-represented racial and ethnic minority was 15.19 years, a non-statistically significant difference.

## Discussion

The present study sought to examine racial, ethnic, and socioeconomic differences in pediatric OSA prevalence and severity, as well as differences in timely OSA detection, among a relatively large, diverse sample with over 1,500 children, over half of whom were on Medicaid insurance. We replicated prior work by showing that children from minority racial and ethnic backgrounds and those from lower SES backgrounds were more likely to be diagnosed with OSA, with greater severity of disease, compared to their peers. Insurance, whether Medicaid or private, was an important factor in predicting the timing and completion of PSG, as those who were uninsured were less likely to complete PSG and tended to complete it at an older age. It is also notable that across racial, ethnic, and socioeconomic backgrounds, only 31.6% of children referred for PSG completed the procedure, and that 47.2% of those who successfully received diagnostic testing were then diagnosed with OSA, highlighting the substantial prevalence of the disorder.

When examining health disparities in the prevalence and severity of pediatric OSA, we found that racially and ethnically under-represented minorities were more likely to be diagnosed with OSA upon completing PSG, had higher AHI values, and had more symptoms associated with OSA, as measured by parent-reports of sleep disordered breathing symptoms, such as snoring, and excessive daytime sleepiness. These findings are consistent with previous research showing that children who are Black or Hispanic tend to have higher rates of OSA (Morton et al., 2001; Goodwin et al., 2003), and higher AHI values (Weinstock et al., 2014). Social and environmental factors likely contribute to observed racial/ethnic disparities in prevalence and severity, such as exposure to environmental toxins in the home (Williamson et al., 2023) or even perinatal exposures, which may influence craniofacial development (Rosen et al., 2003). There is also some evidence to suggest that anatomical or craniofacial differences across races could contribute to differential risk (Dudley and Patel, 2016). Of note, the difference in AHI values that we observed in the present sample across racial/ethnic backgrounds is clinically significant, but the differences in ESS and SDB symptom scores were quite small, though statistically significant. In fact, the difference in ESS (1.45) that we observed between participants who were classified as White and participants who were classified as an under-represented racial/ethnic minority is well below the identified minimally important difference in adults (Patel et al., 2018). The small magnitude of these differences for symptoms associated with OSA suggest that these racial and ethnic disparities should be interpreted with caution, but overall, our findings add to a body of literature that show that Black and Hispanic children tend to experience OSA at a higher rate.

We also found that children with public insurance (i.e., Medicaid) had more severe OSA, with higher AHI values, compared to those with private insurance. Medicaid is provided to families who are living below the federal poverty level; thus insurance type can serve as a useful proxy for SES. The present finding is consistent with previous research showing that when a multitude of SES measures were considered, including maternal education levels and geographic location, children from lower socioeconomic households tended to have higher AHI values (Park et al., 2022). Lower SES families likely experience greater exposure to environmental stressors, such as air pollution, that may contribute to these differences in disease severity (Park et al., 2022). Notably, 40% of children in the United States are estimated to receive Medicaid (Pecha et al., 2021), suggesting that the present findings are also generalizable to a large portion of the population.

The other socioeconomic variable tested, the DCI, was not linked with OSA rates, AHI values, sleep disordered breathing symptoms, or excessive daytime sleepiness. There may be methodological constraints with the DCI estimate, which relies on zip codes to identify neighborhood quality. In some areas, there can be significant SES variability within zip codes, resulting in limited precision. In contrast, Park et al. (2022) accounted for geographic location by sorting zip codes into location type – urban, periurban, rural/remote, and they found that children living in urban areas had more severe OSA with higher AHI values compared to children living in non-urban areas. Such classifications may be useful in future research, as air pollution in urban areas has been shown to cause nasal inflammatory changes, which heightens risk for OSA (Calderon-Garciduenas et al., 1994).

We next examined potential health disparities involved in OSA detection, namely the completion and timing of diagnostic testing for pediatric OSA. Previous research has shown that children who are Black experience longer time to diagnosis (Kilaikode et al., 2018). Families who are historically minoritized by race or ethnicity may experience health care discrimination and mistrust of the healthcare system that may contribute to such delays (Williamson et al., 2023). In contrast to previous work, we did not find racial or ethnic differences in PSG completion rates, PSG timing (i.e., the number of months between the referral date and the service date), or the child's age at the time of PSG. One reason why our sample may not have shown this disparity is that, in the geographic area the study was completed, many of the children identified as URMs tend to live in urban areas closer to the sleep center where the studies were completed. In contrast, White non-Hispanic children were more likely to live in rural areas farther from the sleep center, which could have been a barrier to study completion.

For SES, previous research has shown that children who receive public insurance experience delays in PSG completion (Boss et al., 2015) and are less likely to receive treatment for OSA (Wang et al., 2004), compared to those who are privately ensured. Families from lower SES backgrounds may have limited access to healthcare and may face more barriers to access, such as scheduling difficulties with limited transportation options and work schedule conflicts (Jabbour et al., 2018). We too found significant differences in timely OSA detection—children who were uninsured were the least likely to complete PSG and tended to be older upon completion of the study. This finding reflects a delay in diagnosis among uninsured patients, likely due to the expense of the study, which averages at $3,000 but can be up to $10,000, depending on insurance coverage (Henry et al., 2022). Of note, insurance coverage is not a clear indicator of SES, as individuals with high household income may not have insurance. However, a takeaway that can be drawn from this finding is that lack of insurance appears to be a barrier to timely OSA detection.

Regardless of race, ethnicity, or SES, only 31.6% of the total referred sample completed PSG. This non-completion of diagnostic testing for OSA is consistent with one previous study which showed that, regardless of SES, nearly half of the patients referred for PSG never completed the study (Boss et al., 2015). Non-completion of diagnostic testing may be related to characteristics of our sample. Although the DCI did not emerge as a significant predictor of OSA prevalence, severity, or detection, it provides rich description of the present sample: 48% of the families were classified as from either “at risk” or “distressed” communities, reflecting low high school graduation rates and low median household income, as well as high poverty and unemployment rates. Because nearly half the sample was from such distressed areas, many likely experienced similar barriers to care. Previous research has also highlighted scheduling issues as a barrier to PSG completion (Jean-Louis et al., 2008), which may have similarly affected this sample, across racial, ethnic, and socioeconomic backgrounds. Convenience and perceived necessity of PSG have also been identified in previous research as factors that may affect all families, regardless of their background (Boss et al., 2015). Enhanced efforts to combat these barriers with more transportation options and flexible schedules, as well as enhanced communication through shared decision making about the purpose of the study, will be critical for future research and care provision.

## Limitations and future directions

One notable limitation to the present research is that only a small portion of the sample was uninsured, limiting generalizability of the present findings. Moreover, we only examined sociodemographic predictors of OSA prevalence, severity, and detection. However, delays in detection likely also have systems-level factors that should be examined in future research. For example, future studies could examine the procedures that sleep centers have in place for efficient management of referrals, successful scheduling and completion rates, and ways to support patient understanding and motivation to complete the study.

## Conclusion

By examining electronic health records for over 1,500 children referred for PSG, we showed that children from underrepresented racial/ethnic backgrounds and lower SES backgrounds (as measured by public insurance status) had higher prevalence rates of OSA and worse disease severity. Insurance, whether Medicaid or private, was an important factor in predicting earlier timing and successful completion of PSG, which is essential for diagnosis and treatment of pediatric OSA. Across racial, ethnic, and socioeconomic backgrounds, only 31.6% of the children referred successfully completed PSG. This study adds to a growing literature showing that children from underrepresented racial/ethnic backgrounds or lower socioeconomic backgrounds are more likely to have OSA, which has been associated with emotional and behavioral concerns, negative health outcomes, and poor academic performance. Future research to support efforts to detect and treat OSA to prevent these negative sequalae, especially for the most at-risk children, will be critical.

## Data availability statement

The raw data supporting the conclusions of this article will be made available by the authors, without undue reservation.

## Ethics statement

The studies involving humans were approved by Institutional Review Board of Indiana University of School of Medicine. The studies were conducted in accordance with the local legislation and institutional requirements. Written informed consent for participation was not required from the participants or the participants' legal guardians/next of kin in accordance with the national legislation and institutional requirements.

## Author contributions

MM was the lead author, assisting with data entry, coding, and analysis, as well as manuscript writing and editing. IJ and HA both assisted with data entry and coding as well as initial drafts of the present manuscript. SK assisted with data entry and coding. SH was the principal investigator and assisted with study design, data acquisition, and manuscript writing and editing. All authors contributed to the article and approved the submitted version.
